# Supplementary material for: Allelic effects on KLHL17 expression underlie a pancreatic cancer genome-wide association signal at chr1p36.33
Source: Nat Commun. 2025 Apr 30;16:4055. doi: 10.1038/s41467-025-59109-2 (PMC12044007; doi:10.1038/s41467-025-59109-2)
Supplement: Supplementary file 1 — Supplementary Information [file 41467_2025_59109_MOESM1_ESM.pdf]

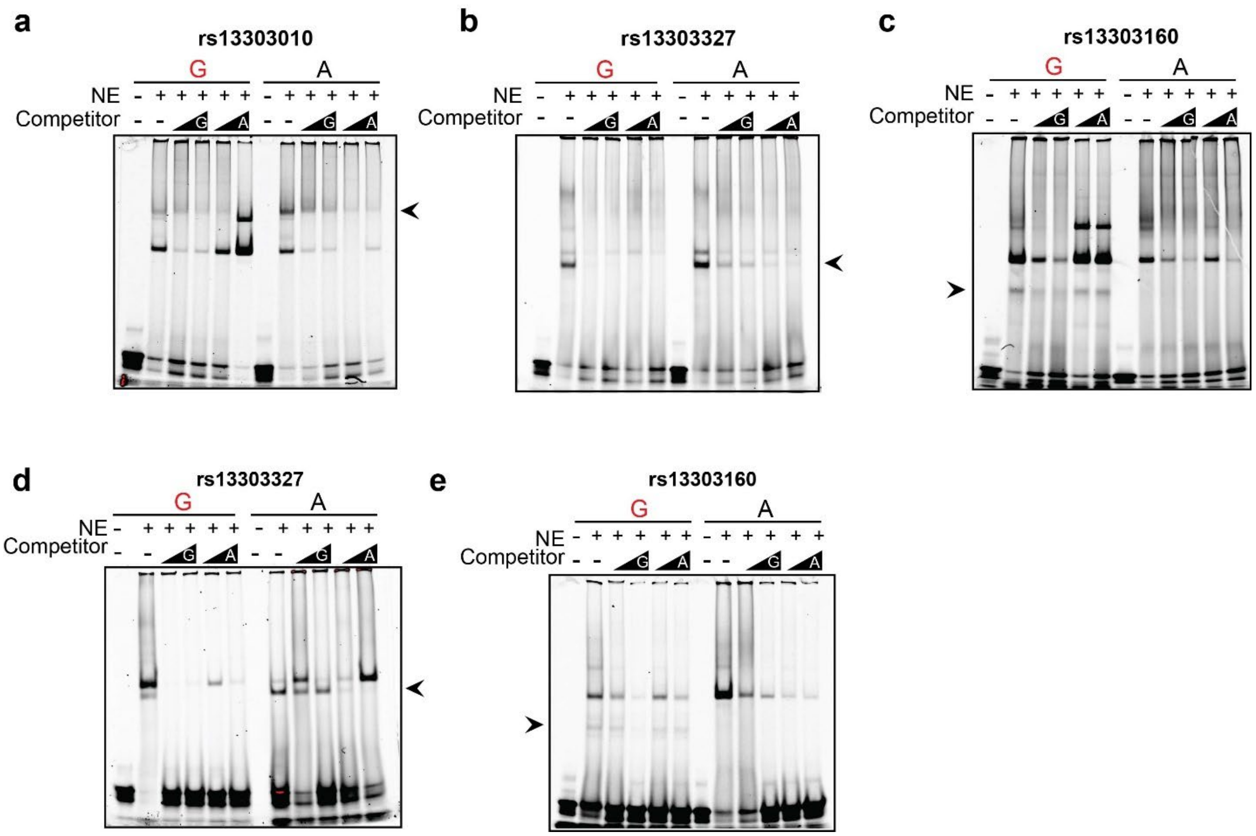

Supplementary Figure 1: Confirmation of allele-preferential binding *in vitro* a-c) Representative EMSA with MIA PaCa-2 nuclear extract and fluorescently labeled oligonucleotides for rs13303010 (n=3 independent replicates), rs13303327 (n=2 independent replicates), rs13303160 (n=2 independent replicates), respectively. Competitor is the same sequence with no fluorescent label in excess (50, 100X); d, e) EMSA with HeLa extract for rs13303327 (n=1) and rs13303160 (n=4 independent replicates), respectively. Risk alleles are indicated in red. Black triangles indicate increasing amounts of the unlabeled competitor (G or A allele). Arrows denote allele-preferential binding.

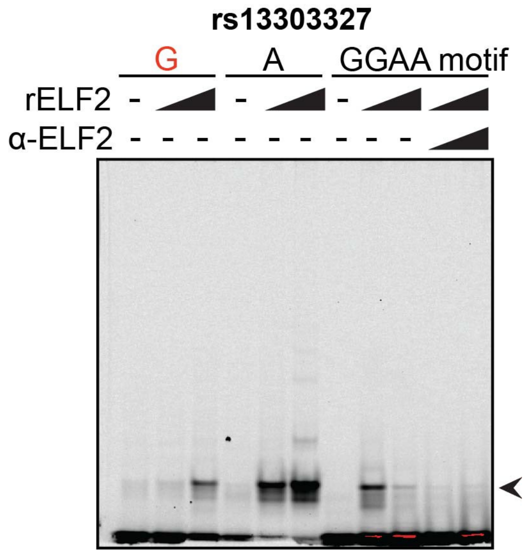

Supplementary Figure 2: Validation of ELF2 binding *in vitro*. An additional representative (of 4 total independent EMSAs) EMSA using recombinant ELF2 and the rs13303327 sequence (see Fig. 3b) or a control sequence with the ELF GGAA motif centered amongst scrambled nucleotides. Increasing amounts of ELF2 recombinant protein were used for each oligo and with the control sequence increasing amounts of ELF2 antibody was included. The risk allele is indicated in red. Black triangles indicate increasing amounts of the recombinant ELF2 protein or ELF2 antibody. The arrow denotes the allele-specific binding bind.

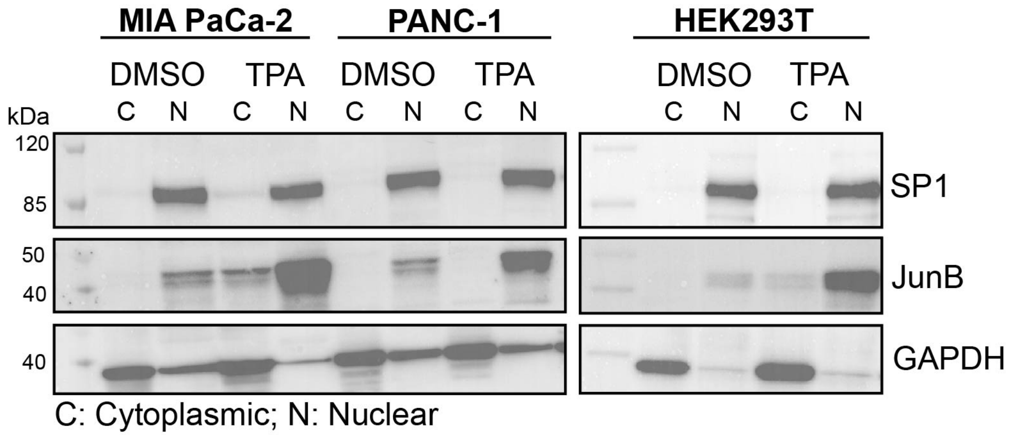

Supplementary Figure 3: Western blot confirmation for induction of AP-1 protein expression with TPA treatment. Western blot analysis using cytoplasmic and nuclear extracts from MIA PaCa-2, PANC-1 and HEK293T cells following 48-hour treatment of cells with DMSO or TPA. Antibodies for SP1 and GAPDH were used as loading controls for nuclear (N) and cytoplasmic (C) extracts, respectively. JunB is used as a proxy for AP-1.

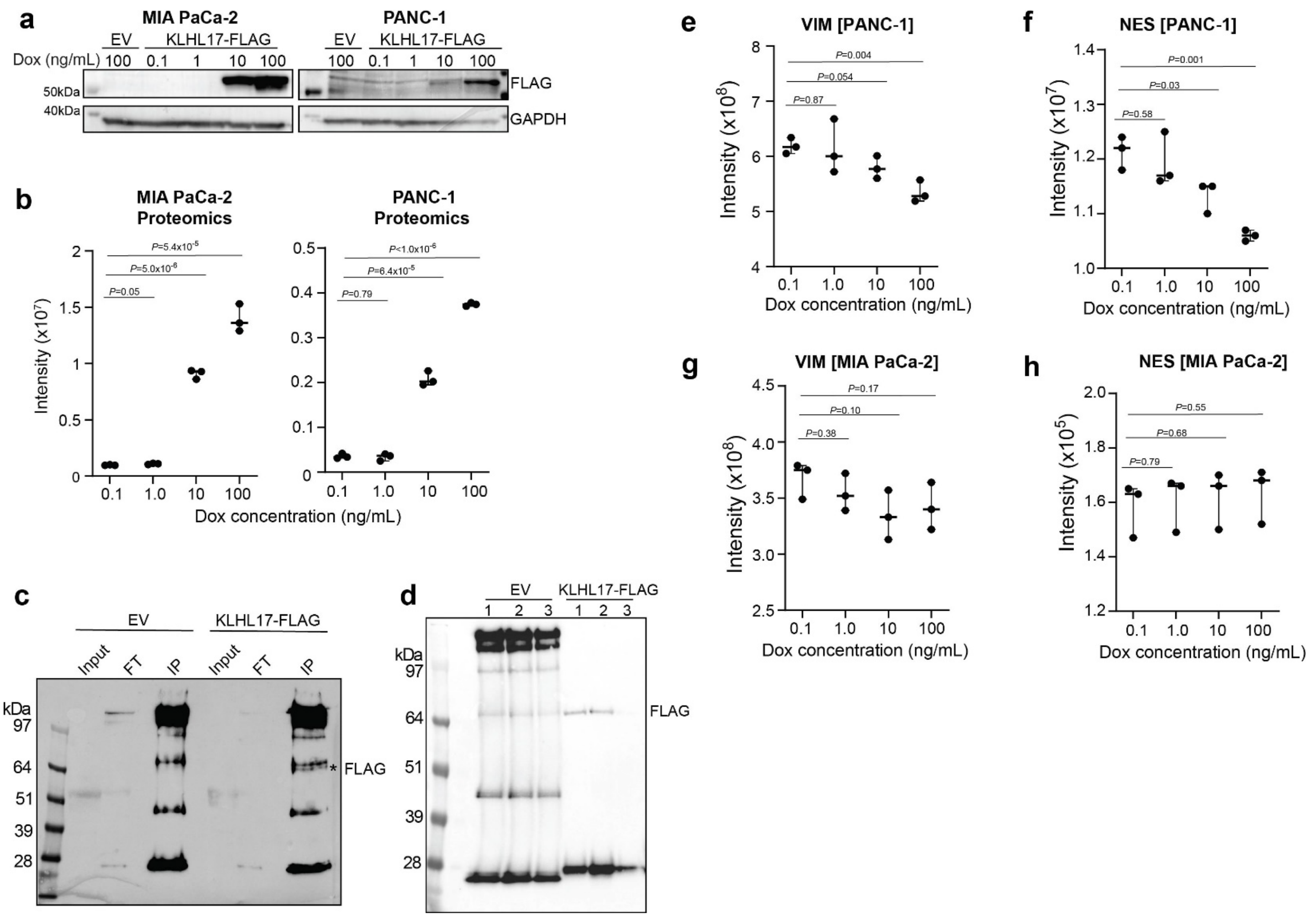

Supplementary Figure 4: Western blot and proteomic analysis of *KLHL17* overexpression and immunoprecipitation. a) Western blot using whole cell lysate from MIA PaCa-2 and PANC-1 cells that were stimulated with increasing amounts of doxycycline for 72 hours to induce KLHL17-FLAG expression. Empty Vector (EV) was used as a negative control for FLAG expression. FLAG antibody was used for detection of induced KLHL17 and GAPDH antibody was used for loading control; b) KLHL17 peptide intensity counts from global proteomic experiments using varying amounts of doxycycline to titrate KLHL17 expression. The median is represented by the horizontal line with 95% confident interval shown as whiskers, n=3 biological replicates per treatment. c) Western blot of the FLAG immunoprecipitation from EV or KLHL17-FLAG expressing PANC-1 cells that was used for a pilot mass-spectrometry analysis to identify co-immunoprecipitated proteins. The blot was probed with a FLAG antibody; d) Western blot of 3 FLAG immunoprecipitation replicates from EV or KLHL17-FLAG expressing PANC-1 cells for mass spectrometry. The blot was probed with a FLAG antibody. e-h) Peptide intensity counts for Vimentin and Nestin in PANC-1 and MIA PaCa-2 cells across different doxycycline concentrations. Lines indicate the median with the 95% confidence intervals shown as whiskers (n=3 biological replicates per treatment). Note the lower peptide intensity values for NES in MIA PaCa-2 cells as compared to PANC-1 cells (y axis counts x10^5 vs. x10^7). Two-tailed, unpaired t-tests were performed relative to 0.1 ng/mL doxycycline.

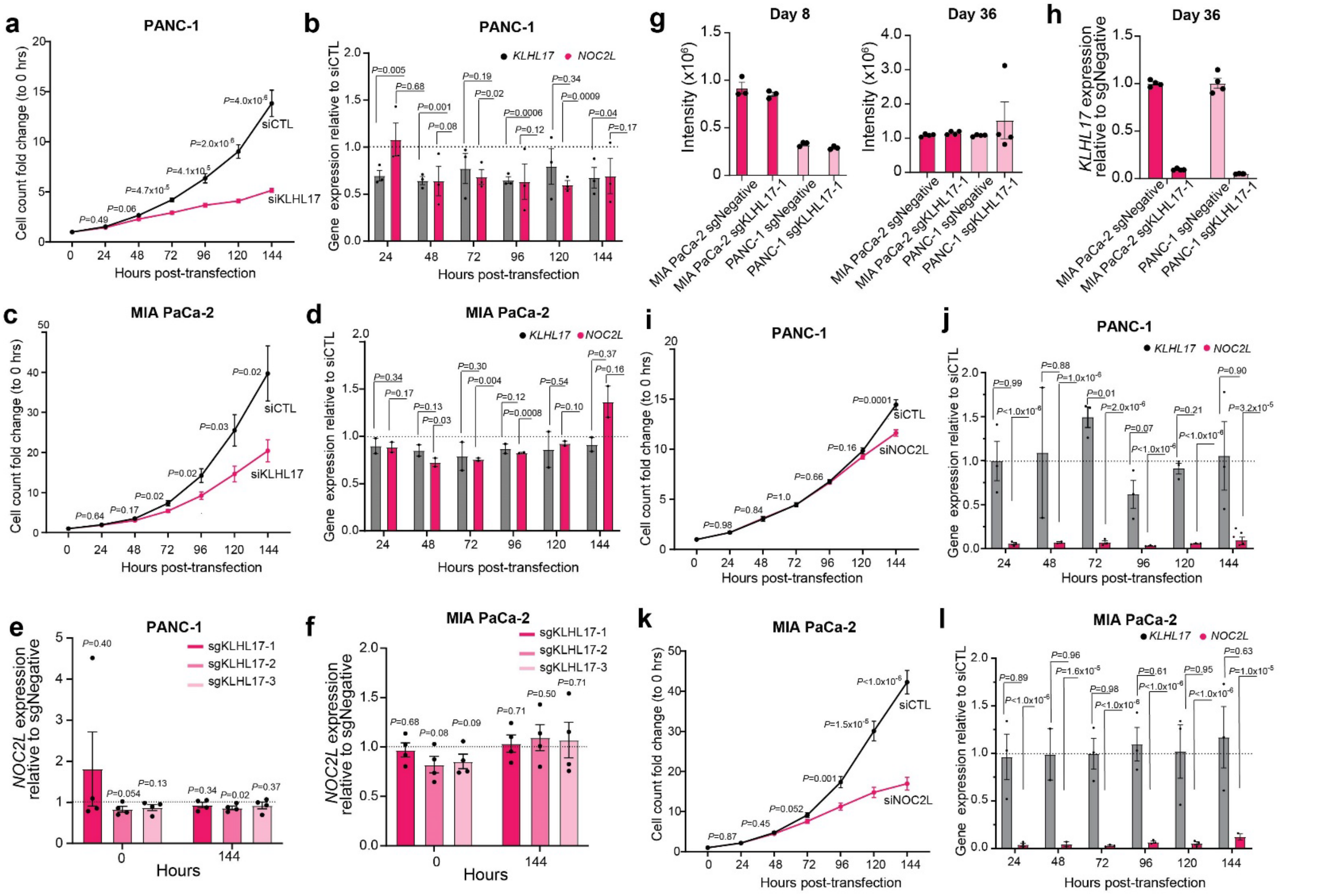

Supplementary Figure 5: *In vitro* growth analysis using siRNA mediated knockdown for *KLHL17* and *NOC2L* a,c) Cell count normalized to 0 hours post-transfection of non-targeting (siCTL, grey) or *KLHL17* siRNA (pink) in the PANC-1 (n=10 biological replicates) and MIA PaCa-2 (n=8 biological replicates) cell lines, respectively; b,d) qPCR analysis of the knockdown efficiency for both *KLHL17* (grey) and nearby *NOC2L* (pink) relative to siCTL and internal *HPRT* control in PANC-1 (n=3 biological replicates) and MIA PaCa-2 (n=2 biological replicates) cells over the course of the growth assay, respectively; e,f) qPCR analysis of *NOC2L* expression in the CRISPRi-mediated *KLHL17* knockdown with three sgRNAs (indicated by different shades of pink) at the beginning and end of the growth analysis in Figure 5 for PANC-1 and MIA PaCa-2 cell lines, respectively. Expression is relative to the sgNegative control (not plotted) and internal control *HPRT*, n=4 biological replicates; g) Peptide intensities for global proteomic analysis at days 8 and 36 in MIA PaCa-2 (dark pink) and PANC-1 (light pink) sgNegative and sgKLHL17 cells. n=3 biological replicates for Day 8 and n=4 biological replicates for Day 36; h) Corresponding qPCR for KLHL17 at Day 36 normalized to the sgNegative and internal *HPRT* control. n=1 biological replicate. Dots represent technical replicates. MIA PaCa-2 cells are denoted by the dark pink bars, and PANC-1 cells by light pink; i,k) Cell count normalized to 0 hours post-transfection of non-targeting (siCTL, grey) or *NOC2L* siRNA (pink) in the PANC-1 (n=12 biological replicates) and MIA PaCa-2 (n=12 biological replicates) cell lines, respectively; j, l) qPCR analysis of the knockdown efficiency for both *KLHL17* (grey) and nearby *NOC2L* (pink) relative to siCTL and internal *HPRT* control in PANC-1 and MIA PaCa-2 cells over the course of the growth assay, respectively, n=3 biological replicates; For all graphs, error bars represent the SEM. Unpaired, two-tailed t-tests were performed.

a

b

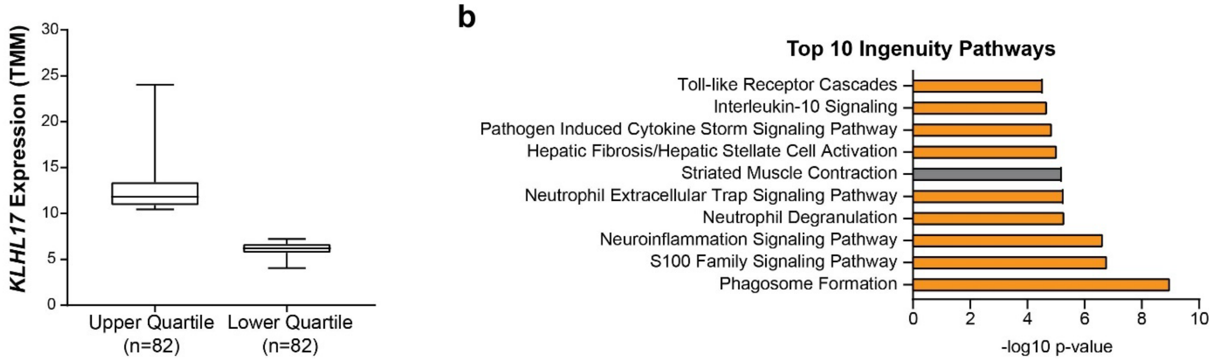

Supplementary Figure 6: *In silico KLHL17* knockdown quartiles and Ingenuity Pathway Analysis (IPA)
a) Boxplot indicating the *KLHL17* expression (TMM) of GTEx Pancreas samples in the upper (75%) and lower (25%) quartiles of samples; b) The top ten most significant pathways from the in silico differential gene expression analysis identified in Ingenuity Pathway Analysis plotted based on the -log10 *P-value*. Orange bars indicate a pathway associated with inflammation.

Supplementary Table 1: Predicted TF motifs disrupted by SNP alleles for either rs13303327 or rs13303160. Alleles 1 and 2 are defined and *P*-values of predicted binding strength are indicated for each allele. The fold-change between the two *P*-values was calculated and used to determine the best predictions.						
**SNP**	**Motif**	**Allele 1**	**Allele 2**	**Allele 1 *P*-value**	**Allele 2 *P*-value**	***P*-value Fold Change**
rs13303327	ETV5	G	A	7.61E-04	1.52E-05	49.96
rs13303327	OLIG2	G	A	2.11E-02	4.53E-04	46.65
rs13303327	TAL1	G	A	6.09E-03	1.60E-04	38.16
rs13303327	TYY1	G	A	1.47E-04	5.98E-06	24.66
rs13303327	ELF5	G	A	6.56E-04	2.66E-05	24.64
rs13303327	ELF2	G	A	2.08E-03	8.70E-05	23.91
rs13303327	NDF1	G	A	4.89E-03	2.06E-04	23.71
rs13303327	NFAT5	G	A	2.00E-03	1.08E-04	18.50
rs13303327	ELF1	G	A	2.27E-03	1.28E-04	17.74
rs13303327	EHF	G	A	2.39E-03	1.35E-04	17.74
rs13303327	NGN2	G	A	7.58E-03	4.49E-04	16.88
rs13303327	GABPA	G	A	2.34E-03	1.45E-04	16.22
rs13303327	ETV7	G	A	2.08E-03	1.36E-04	15.31
rs13303327	ELF3	G	A	1.81E-03	1.31E-04	13.83
rs13303327	ZN816	G	A	3.04E-03	2.57E-04	11.80
rs13303327	E2F6	G	A	1.47E-03	2.05E-04	7.17
rs13303327	ZFP42	G	A	5.65E-04	8.19E-05	6.90
rs13303327	ETS2	G	A	1.70E-03	3.02E-04	5.65
rs13303327	ERG	G	A	3.47E-04	6.65E-05	5.22
rs13303327	PLAG1	G	A	1.13E-04	2.20E-05	5.11
rs13303327	SPI1	G	A	1.92E-03	3.79E-04	5.06
rs13303327	TYY2	G	A	1.19E-05	2.43E-06	4.89
rs13303327	FLI1	G	A	4.58E-04	9.61E-05	4.77
rs13303327	ETS1	G	A	4.77E-04	1.01E-04	4.72
rs13303327	TAF1	G	A	2.36E-05	5.21E-06	4.54
rs13303327	TBX15	G	A	3.28E-05	1.60E-04	0.21
rs13303327	THA11	G	A	4.36E-04	2.27E-03	0.19
rs13303327	ZN770	G	A	3.30E-04	1.77E-03	0.19
rs13303327	ZN263	G	A	2.33E-06	1.28E-05	0.18
rs13303327	ZN143	G	A	2.15E-04	1.33E-03	0.16
rs13303327	EGR1	G	A	1.64E-04	1.03E-03	0.16
rs13303327	RREB1	G	A	2.93E-04	2.16E-03	0.14
rs13303327	Z324A	G	A	1.15E-04	9.57E-04	0.12
rs13303327	ZN140	G	A	1.63E-04	1.61E-03	0.10
rs13303327	ZN320	G	A	7.57E-05	7.54E-04	0.10
rs13303327	ZN281	G	A	7.57E-05	7.61E-04	0.10
rs13303327	ZNF76	G	A	5.79E-05	5.82E-04	0.10
rs13303327	WT1	G	A	7.60E-06	8.20E-05	0.09
rs13303327	KLF15	G	A	4.99E-08	5.49E-07	0.09
rs13303327	EGR2	G	A	5.84E-05	7.54E-04	0.08
rs13303327	ZN263	G	A	9.79E-06	1.47E-04	0.07
rs13303327	PURA	G	A	1.06E-06	6.52E-05	0.02
rs13303160	FOSL1	G	A	4.32E-04	4.94E-06	87.31
rs13303160	JUND	G	A	9.47E-04	2.28E-05	41.63
rs13303160	BATF	G	A	1.08E-02	2.65E-04	40.95

rs13303160	FOSB	G	A	1.18E-03	2.93E-05	40.11
rs13303160	FOSL2	G	A	8.16E-04	2.15E-05	37.93
rs13303160	JUNB	G	A	8.00E-04	2.47E-05	32.36
rs13303160	JUN	G	A	8.40E-04	2.68E-05	31.37
rs13303160	ZFX	G	A	1.90E-03	7.88E-05	24.15
rs13303160	FOS	G	A	5.73E-04	2.74E-05	20.86
rs13303160	BACH2	G	A	8.84E-04	8.59E-05	10.29
rs13303160	MAFK	G	A	2.19E-03	2.33E-04	9.38
rs13303160	BACH1	G	A	1.80E-04	2.85E-05	6.30
rs13303160	ZN554	G	A	6.28E-04	1.11E-04	5.65
rs13303160	NFE2	G	A	3.72E-04	7.80E-05	4.77
rs13303160	ZN329	G	A	1.08E-03	2.35E-04	4.58
rs13303160	PAX2	G	A	1.68E-04	4.00E-05	4.19
rs13303160	MBD2	G	A	4.22E-06	2.85E-05	0.15
rs13303160	ATF6A	G	A	3.91E-04	4.34E-03	0.09
rs13303160	THAP1	G	A	1.09E-04	1.34E-03	0.08
rs13303160	HEY2	G	A	1.58E-04	1.96E-03	0.08
rs13303160	HES7	G	A	1.08E-04	1.53E-03	0.07
rs13303160	HES5	G	A	4.58E-04	7.07E-03	0.06
rs13303160	HEY1	G	A	2.65E-04	7.97E-03	0.03

Supplementary Table 2: Sequences of oligonucleotides used for EMSAs. The IRDye700 and unlabeled oligonucleotides are the same.

	**Forward Oligo**	**Reverse Complement Oligo**
**rs13303010 REF**	CCGGGAGGCCAAATC**G**GCCCTCGGACCCGCG	CGCGGGTCCGAGGGC**C**GATTTGGCCTCCCGG
**rs13303010 ALT**	CCGGGAGGCCAAATC**A**GCCCTCGGACCCGCG	CGCGGGTCCGAGGGC**T**GATTTGGCCTCCCGG
**rs13303160 REF**	AGTCACCGGTGACTC**G**GGCCGGCCAGAGTTT	AAACTCTGGCCGGCC**C**GAGTCACCGGTGACT
**rs133030160 ALT**	AGTCACCGGTGACTC**A**GGCCGGCCAGAGTTT	AAACTCTGGCCGGCC**T**GAGTCACCGGTGACT
**rs13303327 REF**	CTTCCCAGAGGAGGA**G**GATGGCGGGGCCTGG	CCAGGCCCCGCCATC**C**TCCTCCTCTGGGAAG
**rs13303327 ALT**	CTTCCCAGAGGAGGA**A**GATGGCGGGGCCTGG	CCAGGCCCCGCCATC**T**TCCTCCTCTGGGAAG
**ELF motif**	GTCATGACGAACCCGGAAGTAGTCATGACGA	TCGTCATGACTACTTCCGGGTTCGTCATGAC

Supplementary Table 3: Gene Block (gBlock) sequences used for luciferase assays. Assays were completed using the forward and reverse orientations of the sequence. Sequence lengths were determined based on the epigenomic annotations. For rs13303327, gBlocks could not be synthesized and was cloned from a HapMap subject heterozygous for the SNP using the primers listed.

	**gBlock Sequences 5' - 3'**
**rs13303010 Forward Ref**	TCA GCTAGC GCAGGAGTCACAGCTGCCCGCACGCCCAGCTCGCCCCAGCCCCGCTGAGAGGAGCAAGAAAAGCCCCCTTGGATA CAGACACCCACCGGGAGGCCAAATCGGCCCTCGGACCCGCGGCTTACCTCTTGCGGCTCCCCGCAGCTGCCATGACACCAACCCG AAGCGTGCACCCCACTTCCGGCCCCAGAATGCCGCGCGGCT AAGCTT CTG
**rs13303010 Forward Alt**	TCAGCTAGCGCAGGAGTCACAGCTGCCCGCACGCCCAGCTCGCCCCAGCCCCGCTGAGAGGAGCAAGAAAAGCCCCCTTGGATAC AGACACCCACCGGGAGGCCAAATCAGCCCTCGGACCCGCGGCTTACCTCTTGCGGCTCCCCGCAGCTGCCATGACACCAACCCGA AGCGTGCACCCCACTTCCGGCCCCAGAATGCCGCGCGGCT AAGCTTCTG
**rs13303010 Reverse Ref**	TCAGCTAGCAGCCGCGCGGCATTCTGGGGCCGGAAGTGGGGTGCACGCTTCGGGTTGGTGTCATGGCAGCTGCGGGGAGCCGCA AGAGGTAAGCCGCGGGTCCGAGGGCCGATTTGGCCTCCCGGTGGGTGTCTGTATCCAAGGGGGCTTTTCTTGCTCCTCTCAGCGG GGCTGGGGCGAGCTGGGCGTGCGGGCAGCTGTGACTCCTGC AAGCTTCTG
**rs13303010 Reverse Alt**	TCAGCTAGCAGCCGCGCGGCATTCTGGGGCCGGAAGTGGGGTGCACGCTTCGGGTTGGTGTCATGGCAGCTGCGGGGAGCCGCA AGAGGTAAGCCGCGGGTCCGAGGGCTGATTTGGCCTCCCGGTGGGTGTCTGTATCCAAGGGGGCTTTTCTTGCTCCTCTCAGCGG GGCTGGGGCGAGCTGGGCGTGCGGGCAGCTGTGACTCCTGC AAGCTTCTG
**rs13303160 Forward Ref**	TCAGCTAGCTGCGGTGGCTTTGGCCGCCGTCTCCTGTGCTGGAACTCCTGCCTCAGCCCTCCCTGCAGTCACCGGTGACTCGGGC CGGCCAGAGTTTAGATGGAAACAGGATGTGTGGGCACGTTGTCCCGGGGGGCCTGGAAGGTCGCCCC GGAAGCTTCTG
**rs13303160 Forward Alt**	TCAGCTAGCTGCGGTGGCTTTGGCCGCCGTCTCCTGTGCTGGAACTCCTGCCTCAGCCCTCCCTGCAGTCACCGGTGACTCAGGC CGGCCAGAGTTTAGATGGAAACAGGATGTGTGGGCACGTTGTCCCGGGGGGCCTGGAAGGTCGCCCC GGAAGCTTCTG
**rs13303160 Reverse Ref**	TCAGCTAGCGGGGCGACCTTCCAGGCCCCCCGGGACAACGTGCCCACACATCCTGTTTCCATCTAAACTCTGGCCGGCCCGAGTC ACCGGTGACTGCAGGGAGGGCTGAGGCAGGAGTTCCAGCACAGGAGACGGCGGCCAAAGCCACCG AAGCTTCTG
**rs13303160 Reverse Alt**	TCAGCTAGCGGGGCGACCTTCCAGGCCCCCCGGGACAACGTGCCCACACATCCTGTTTCCATCTAAACTCTGGCCGGCCTGAGTC ACCGGTGACTGCAGGGAGGGCTGAGGCAGGAGTTCCAGCACAGGAGACGGCGGCCAAAGCCACCG AAGCTTCTG
	Primers for amplification of rs13303327 from HapMap (165bp amplicon + vector overhang)
**rs13303327 Forward Orientation Forward Primer**	TCAGCTAGCGGAAGAAGGAAGGCGAGACCTAG
**rs13303327 Forward Orientation Reverse Primer**	CAGAAGCTTGCCTCGCCCTCCTCCTC
**rs13303327 Reverse**	CAGAAGCTTGGAAGAAGGAAGGCGAGACCTAG

**Orientation Forward Primer**	
**rs13303327 Reverse Orientation Reverse Primer**	TCAGCTAGCGCCTCGCCCTCCTCCTC

Supplementary Table 4: Sequences for the gRNAs used in CRISPRi experiments. Guide sequences were determined using the CRISPOR feature on the UCSC Genome Browser.

Guide Name	Forward Strand (5'-3')	Reverse Complement (5'-3')
**sgKLHL17-1**	TTGACGGACGCGGAGACTGCCGGGTTTAAGAGC	TTAGCTCTTAAACCCGGCAGTCTCCGCGTCCGTCAACAAG
**sgKLHL17-2**	TTGCTCCGCGTCCGTTAAGCCCGGTTTAAGAGC	TTAGCTCTTAAACCGGGCTTAACGGACGCGGAGCAACAAG
**sgKLHL17-3**	TTGGTCCTCCGCGAATCGGCGGTGTTTAAGAGC	TTAGCTCTTAAACACCGCCGATTCGCGGAGGACCAACAAG
**sgNegative**	TTGTGGCTAGCAACATCTCGACAGTTTAAGAGC	TTAGCTCTTAAACTGTCGAGATGTTGCTAGCCACAACAAG

Supplementary Table 5: Sequences of primers used for ChIP-qPCR. Controls for ELF2 came from a K562 GFP-ELF2 ChIP-seq (GSE177468). JunB/D positive control was determined from a JunB ChIP-seq in CFPAC1 cells (GSE119930). The negative control is from a quiescent region on chromosome 1 (4176991-4180055 (hg19))

Primer Set	Forward 5' - 3'	Reverse 5' - 3'
**rs1303327_PS1**	GAAGGAAGGCGAGACCTAGG	TCCGAGAAGCCCCCCTAGG
**rs13303327_PS2**	AGAAGGAAGGCGAGACCTA	CGTTGGACGCGGATTCTT
**rs13303327_PS3**	GGGTCCCATTCGACTTCTTG	TAGGTCTCGCCTTCCTTCTT
**ELF2 Positive Control (PYGO2)**	AGGCGTAGCGTCTCGTCCG	GAGCTGCAGCAACCACAAAGTG
**ELF2 Positive Control (TBP)**	GTGACCTATGCTCACACTTCTC	GAGTACAATCTGTTACCTGGGTC
**rs13303160_PS1**	GAGTCCCCTTAAGCCTTGGG	GAGTTCCAGCACAGGAGACG
**rs13303160_PS2**	TCTCCTGTGCTGGAACTCCT	CCTTTCTGGAAGAGGCCTGG
**rs13303160_PS3**	TTAAGCCTTGGGGACCCTGA	GCCCACACATCCTGTTTCCA
**Positive Control**	ATGCACGAGGCCTTTGAGAA	CAGGCAGTTCCTGTTGCCTA
**Negative Control**	GAACTCGAAAGGCACCAGGA	AAGGCCCTCTGGATGGATCT
