# Supplementary material for: Allelic effects on KLHL17 expression underlie a pancreatic cancer genome-wide association signal at chr1p36.33
Source: Nat Commun. 2025 Apr 30;16:4055. doi: 10.1038/s41467-025-59109-2 (PMC12044007; doi:10.1038/s41467-025-59109-2)
Supplement: Supplementary file 2 — Description of Additional Supplementary Files [file 41467_2025_59109_MOESM2_ESM.pdf]

Description of Additional Supplementary Files

Supplementary Data 1: Dataset of global protein expression (peptide intensity values) for all proteins identified in the IP-MS data
