# Supplementary material for: Allelic effects on KLHL17 expression underlie a pancreatic cancer genome-wide association signal at chr1p36.33
Source: Nat Commun. 2025 Apr 30;16:4055. doi: 10.1038/s41467-025-59109-2 (PMC12044007; doi:10.1038/s41467-025-59109-2)
Supplement: Supplementary file 4 — Reporting Summary [file 41467_2025_59109_MOESM4_ESM.pdf]

nature portfolio

Corresponding author(s): Laufey Amundadottir, Katelyn Connelly

Last updated by author(s): Mar 26, 2025

Reporting Summary

Nature Portfolio wishes to improve the reproducibility of the work that we publish. This form provides structure for consistency and transparency in reporting. For further information on Nature Portfolio policies, see our Editorial Policies and the Editorial Policy Checklist.

Statistics

For all statistical analyses, confirm that the following items are present in the figure legend, table legend, main text, or Methods section.

n/a	Confirmed	
☐	☒	The exact sample size (*n*) for each experimental group/condition, given as a discrete number and unit of measurement
☐	☒	A statement on whether measurements were taken from distinct samples or whether the same sample was measured repeatedly
☐	☒	The statistical test(s) used AND whether they are one- or two-sided<br>*Only common tests should be described solely by name; describe more complex techniques in the Methods section.*
☐	☒	A description of all covariates tested
☒	☐	A description of any assumptions or corrections, such as tests of normality and adjustment for multiple comparisons
☐	☒	A full description of the statistical parameters including central tendency (e.g. means) or other basic estimates (e.g. regression coefficient) AND variation (e.g. standard deviation) or associated estimates of uncertainty (e.g. confidence intervals)
☐	☒	For null hypothesis testing, the test statistic (e.g. *F*, *t*, *r*) with confidence intervals, effect sizes, degrees of freedom and *P* value noted<br>*Give P values as exact values whenever suitable.*
☒	☐	For Bayesian analysis, information on the choice of priors and Markov chain Monte Carlo settings
☒	☐	For hierarchical and complex designs, identification of the appropriate level for tests and full reporting of outcomes
☒	☐	Estimates of effect sizes (e.g. Cohen's *d*, Pearson's *r*), indicating how they were calculated

Our web collection on statistics for biologists contains articles on many of the points above.

Software and code

Policy information about availability of computer code

Data collection	NA
Data analysis	SNPTESTv2.5.4-beta3 was used for the GWAS analysis of the UK Biobank data. Metal (03/25/2011) was used for the meta-analysis of the GWAS summary statistics. SuSiE (v.012.35) was used in R-studio (v. 2022.02.3+492) for the fine-mapping analysis, Plink (v1.07) was used to generate the required LD matrix. EdgeR (v.3.38.1) was used in R-studio for differential gene expression analysis. coloc (v.4) was used in RStudio for the colocalization analysis.

For manuscripts utilizing custom algorithms or software that are central to the research but not yet described in published literature, software must be made available to editors and reviewers. We strongly encourage code deposition in a community repository (e.g. GitHub). See the Nature Portfolio guidelines for submitting code & software for further information.

Data

Policy information about availability of data

All manuscripts must include a data availability statement. This statement should provide the following information, where applicable:

- Accession codes, unique identifiers, or web links for publicly available datasets
- A description of any restrictions on data availability
- For clinical datasets or third party data, please ensure that the statement adheres to our policy

The proteomics data generated in this study have been deposited in the MassIVE database under accession code #MSV000096025 [https://massive.ucsd.edu/ProteoSAFe/private-dataset.jsp?task=e357b08275e34f89b280e716ccd20d8f]. The PDAC GWAS data is available under controlled access for data use limitation and

can be requested through dbGaP: phs000206.v6.p3 [https://www.ncbi.nlm.nih.gov/projects/gap/cgi-bin/study.cgi?study_id=phs000206.v6.p3], phs000648.v1.p1 [https://www.ncbi.nlm.nih.gov/projects/gap/cgi-bin/study.cgi?study_id=phs000648.v1.p1]. UK Biobank data is available through the UK Biobank (https://www.ukbiobank.ac.uk/). Source data are provided with this paper.

Research involving human participants, their data, or biological material

Policy information about studies with human participants or human data. See also policy information about sex, gender (identity/presentation), and sexual orientation and race, ethnicity and racism.

Reporting on sex and gender	No reporting on sex or gender, all analyses were performed for both females and males combined.
Reporting on race, ethnicity, or other socially relevant groupings	No reporting on race, ethnicity or social groupings.
Population characteristics	European ancestry pancreatic ductal adenocarcinoma cases and unaffected control subjects. The GWAS was performed in European ancestry individuals recruited in the US, Canada and Europe. Age and sex were used as covariates.
Recruitment	Recruitment for the GWAS study was performed via cohort and case-control studies. The disease under study is Pancreatic Ductal Adenocarcinoma (PDAC) – ICD10 codes (C25 with the exception of C25.4).
Ethics oversight	All participating studies have institutional IRB approvals and the NCI part of the study has NIH Special Study IRB approval.

Note that full information on the approval of the study protocol must also be provided in the manuscript.

Field-specific reporting

Please select the one below that is the best fit for your research. If you are not sure, read the appropriate sections before making your selection.

[x] Life sciences [ ] Behavioural & social sciences [ ] Ecological, evolutionary & environmental sciences

For a reference copy of the document with all sections, see nature.com/documents/nr-reporting-summary-flat.pdf

Life sciences study design

All studies must disclose on these points even when the disclosure is negative.

Sample size	Sample sizes were chosen based on the sensitivity and variability of the experiments, but sample size calculations were not performed. More replicates were included if after three biological replicates a trend was clear but had not reached significance.
Data exclusions	No data was excluded.
Replication	To verify the reproducibility, experiments were repeated multiple times on different days and with different cell lines or lysate to ensure reproducibility. For several experiments, the results were reproduced by different people (rs13303160 luciferase, siRNA growth analysis, CRISPRi knockdown). All data is presented. In some cases, ChIP-qPCR using the primers did not show enrichment in every replicate, but all replicates are reported. This lack of enrichment in the qPCR could be a result of numerous technical reasons (primers, IP efficiency, shearing quality, etc.), however, using a TaqMan genotyping assay from the same experiment did result in enrichment each time.
Randomization	No randomization was necessary for the association studies or in vitro experiments. For cell experiments, cells were plated and at the time of transfection, the wells treated with the control were randomly selected. For ChIP-qPCR, sheared chromatin was divided evenly between control and target IP. Participants in the GWAS were classified by disease status (case or control) as that is what was needed for the association tests.
Blinding	No blinding of data was necessary for the analyses performed. Association tests were performed in regards to disease status.

Reporting for specific materials, systems and methods

We require information from authors about some types of materials, experimental systems and methods used in many studies. Here, indicate whether each material, system or method listed is relevant to your study. If you are not sure if a list item applies to your research, read the appropriate section before selecting a response.

Materials & experimental systems

n/a	Involved in the study
☐	☒ Antibodies
☐	☒ Eukaryotic cell lines
☒	☐ Palaeontology and archaeology
☒	☐ Animals and other organisms
☐	☒ Clinical data
☒	☐ Dual use research of concern
☒	☐ Plants

Methods

n/a	Involved in the study
☒	☐ ChIP-seq
☒	☐ Flow cytometry
☒	☐ MRI-based neuroimaging

Antibodies

Antibodies used

For EMSA: ELF2 (ab28726, Abcam; 12499-1-AP, Proteintech Rosemont, IL), JunB (C37F9, Cell Signaling Technologies, Danvers, MA), JunD (D17G2, Cell Signaling Technologies, Danver, MA). For Western Blot and Immunoprecipitation: JunB (1:1000, C37F9, Cell Signaling Technologies, Danver, MA), JunD (1:1000, D17G2, Cell Signaling Technologies, Danver ,MA), FLAG (1:1000, F1804, Millipore Sigma, Burlington, MA), KLHL17 (1:500, PA5-56689 Thermo Fisher Scientific, Waltham, MA); 1:500, HPA031251, Millipore Sigma), GAPDH (1:1000, ab125247, Abcam, Waltham, MA), SP1 (1:000 ab13370, Abcam); mouse anti-rabbit light chain specific antibody HRP (1:5000, C840Z39 Jackson ImmunoResearch, West Grove, PA); donkey anti-mouse secondary HRP (1:5000, ab7061, Abcam, Waltham, MA), donkey anti-rabbit secondary HRP (1:5000, ab205722, Abcam, Waltham, MA); For ChIP: ELF2 (4 ug, 12499-1-AP, Proteintech, Rosemont, IL), JunB (10 uL, C37F9,Cell Signaling Technologies, Danver, MA), JunD (4 ug, 720035, Invitrogen, Waltham, MA), Rabbit IgG (4 ug, 2729S, Cell Signaling Technologies, Danver, MA). Immunofluorescence: KLHL17 (1ug/mL, HPA031251, Millipore Sigma, Burlington, MA), AlexaFluor647 (1:1000, A-31573, Thermo Fisher Scientific, Waltham, MA)

Validation

All antibodies used were validated for the appropriate method by the manufacturer and details can be found on the respective vendors' website. Additionally, the KLHL17 Sigma antibody is a Human Protein Atlas validated antibody.

Eukaryotic cell lines

Policy information about cell lines and Sex and Gender in Research

Cell line source(s)

MIA PaCa-2 (CRM-CRL-1420), PANC-1 (CRL-1469), Hs766T (HTB-134), and HEK293T (CRL-3216) were purchased from ATCC. SW1990 cell line was a gift from Dr. Jaiswal Kshama.

Authentication

Cell lines were also tested for authentication with a panel of short tandem repeats (STRs) via the Identifiler kit (Life Technologies, Carlsbad, CA) and compared with ATCC and DSMZ (German Collection of Microorganisms and Cell Cultures – https://www.dsmz.de/) STR profile datasets. All cell lines with profiles in the databases matched and those not with profiles in this database matched earlier passages of these cell lines in use in our laboratory.

Mycoplasma contamination

Cell lines were routinely tested for mycoplasma and were always found to be negative

Commonly misidentified lines
(See ICLAC register)

None were used.

Clinical data

Policy information about clinical studies

All manuscripts should comply with the ICMJEguidelines for publication of clinical research and a completedCONSORT checklist must be included with all submissions.

Clinical trial registration

NA

Study protocol

NA

Data collection

NA

Outcomes

NA

Plants

Seed stocks	*Report on the source of all seed stocks or other plant material used. If applicable, state the seed stock centre and catalogue number. If plant specimens were collected from the field, describe the collection location, date and sampling procedures.*
Novel plant genotypes	*Describe the methods by which all novel plant genotypes were produced. This includes those generated by transgenic approaches, gene editing, chemical/radiation-based mutagenesis and hybridization. For transgenic lines, describe the transformation method, the number of independent lines analyzed and the generation upon which experiments were performed. For gene-edited lines, describe the editor used, the endogenous sequence targeted for editing, the targeting guide RNA sequence (if applicable) and how the editor was applied.*
Authentication	*Describe any authentication procedures for each seed stock used or novel genotype generated. Describe any experiments used to assess the effect of a mutation and, where applicable, how potential secondary effects (e.g. second site T-DNA insertions, mosiacism, off-target gene editing) were examined.*
